# Automation in the fish industry

**DOI:** 10.1093/af/vfac020

**Published:** 2022-04-30

**Authors:** Hildur Einarsdóttir, Bergur Guðmundsson, Valdimar Ómarsson

**Affiliations:** Marel, Garðabær,Iceland

**Keywords:** artificial intelligence, automation, fish processing, robotics

**Implications**

• The fish industry has historically been a labor-intensive field, requiring skilled staff to process whole fish into consumable products.

• The introduction of automation in food production has been at a slower pace compared with conventional manufacturing industries such as automotive due to the organic variation present in food products.

• Recent advances in automated inspection, artificial intelligence, and robotics are transforming the food production industry, introducing new automation capabilities that can potentially increase throughput and yield.

## Introduction

Historically food processing has been labor-intensive, with the seafood industry being no exception. Until recently, the biggest share of seafood production comes from wild harvest with a large variety of species and different raw material conditions, subject to natural fluctuation and handling of the catch. Mathiesen (2012) stated that a considerable number of the world population have their employment linked to the fish industry in some way, be that primary production, processing, packaging, distribution, etc. The production of seafood from raw material to consumable products requires skilled people for filleting, trimming, peeling, and visual parasite and quality control. The largest wild fish resources are often located in remote or scarcely populated areas, and the same applies to some of the best salmon aquaculture sites. The fish industry, therefore, often deals with significant challenges in finding people willing to work in the processing plants.

The jobs are often difficult and repetitive, and even dangerous. The quality of seafood deteriorates quickly, especially with increased temperature. For this reason, the product is always kept as cold as possible. The work environment is, therefore, cold and humid, making it difficult for the workers to stay in for the duration of an entire shift or even longer as is often the case on board factory vessels and during seasonal peaks in the wild harvest. With the COVID-19 pandemic, food producers are finding it difficult to recruit people due to social distancing and border travel restrictions (Aday and Aday, 2020; Minahal et al., 2020; Hailu, 2021). High-income regions often rely on migrant workers, creating challenges maintaining the supply chain.

With poor access to manual labor, great focus has been placed on automating food production. Through recent decades, automation within fish production is becoming more prevalent, making it one of the most high-tech automated sectors within the protein industry alongside poultry (Komlatsky et al., 2019). Significant advances in the last decade are automated de-heading (Buckingham et al., 2001) and filleting machines (Andersen and Magnusson, 2002).

Automation has enabled an enormous increase in line speed and throughput. In the 1990s, pelagic fish factories processed and froze 150 kg of fish per worker per day. Today, factories can process and freeze significantly beyond 1,500 kg per person per day (Sigurðardóttir, 2018), with reports as high as 15,000 kg per person per day (Vigfússon and Gestsson, 2016). In the most automated fish factories, the yield and utilization of fish are estimated to have improved from around 60% to 80% in the past decade.

**Figure 1.**
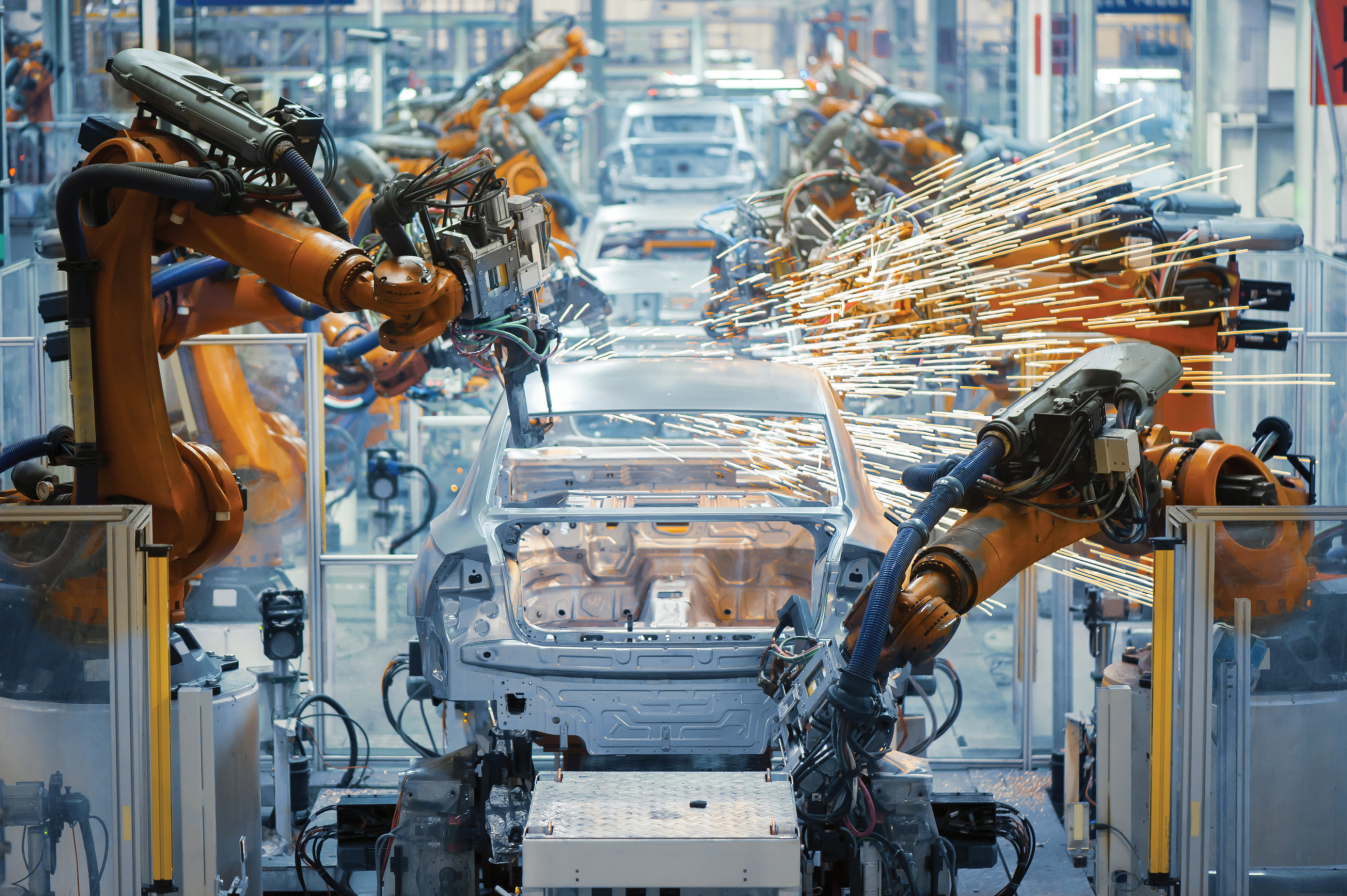
An automotive factory floor where robots are seen spot-welding car bodies (Jenson, 2021).

Many processing steps still, however, rely on manual labor. These are tasks such as trimming of fillets and quality inspection. Although these tasks may seem trivial, in reality, they are quite challenging. To elaborate further, an example from the automotive industry is given. In an automotive factory, almost every step in assembling a car is automated. Figure 1 shows an automotive factory floor where a number of robotic arms are placed on an assembly line, each specifically programmed to perform a limited and well-defined task. Due to the static (and rigid) nature of every component, the variation between specific car parts is limited, making it simple to automate for tasks such as the attachment of a door and the car frame. In contrast, each fish varies in size, weight, color, and physical shape. The state of rigor mortis (a postmortem change resulting in the stiffening of the body muscles) also varies between fish, based on multiple variables such as pre-rigor stress and environmental temperature. The differences in raw material core temperature will accelerate or decelerate the rigor cycle of each fish. A machine that automates the filleting step needs to be able to cope with these variations and adjust accordingly for every fish to reach an acceptable speed and yield. Another significant difference between manufacturing a car and processing a fish is the fact that the car is being assembled, while the fish is being taken apart. Furthermore, in fish processing, traceability from raw product intake to end consumable product is necessary in many cases in order to validate the country of origin and validate the quality and remaining shelf life. Finally, gathering the weight of each piece and product quality information is important to give food producers insight to optimize their process.

## Fish Processing

**Figure 2.**
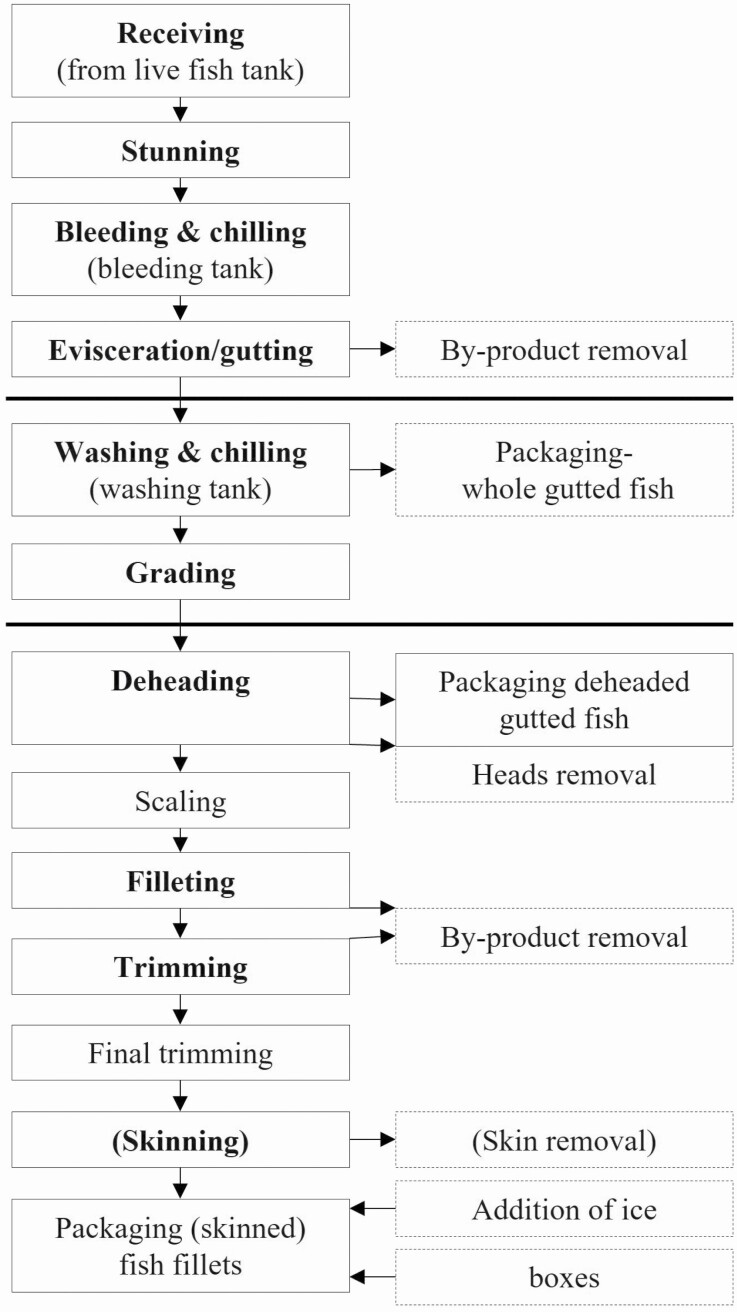
An example of a fish processing pipeline from receiving to packaging (figure based on Vidaček and Bugge, 2016).

Figure 2 provides an overview of a fish production process, illustrating the key concepts. The detailed steps and their order can vary depending on fish species, and whether the fish is wild-caught or farmed. After capturing the fish, it is transported to processing facilities, either on board a fishing vessel or to a land-based facility. If the fish is wild-caught, an initial sorting of different species is required, and sometimes the fish is graded based on size, weight, and quality. For a farmed fish, a stunning process takes place prior to additional processing steps. The fish is then bled, which is a necessary step as blood is a good nutrition for bacteria, and the presence of blood can reduce the shelf life and value of the catch. Bleeding is performed in dedicated tanks with clean circulating seawater. After bleeding, the fish is chilled, followed by evisceration and gutting. Gutting is the process of cutting the belly open, removing internal organs, and making sure the body cavity is clean of blood residual and other excess residues. During the gutting process, byproducts can be extracted such as the liver for fish oil production. The fish can then be further processed depending on what the end product should be; in some cases, the fish will be packaged whole, while in others it will undergo a de-heading process and even filleted, trimmed, and further portioned. The exact end product also dictates any additional processing steps needed (e.g., sushi portions, fresh steaks, smoked salmon slices, fish cakes, and nuggets). In any case, the packaging of the product is required for transportation to retail, food service, or consumer.

## Recent Automation Advances

**Figure 3.**
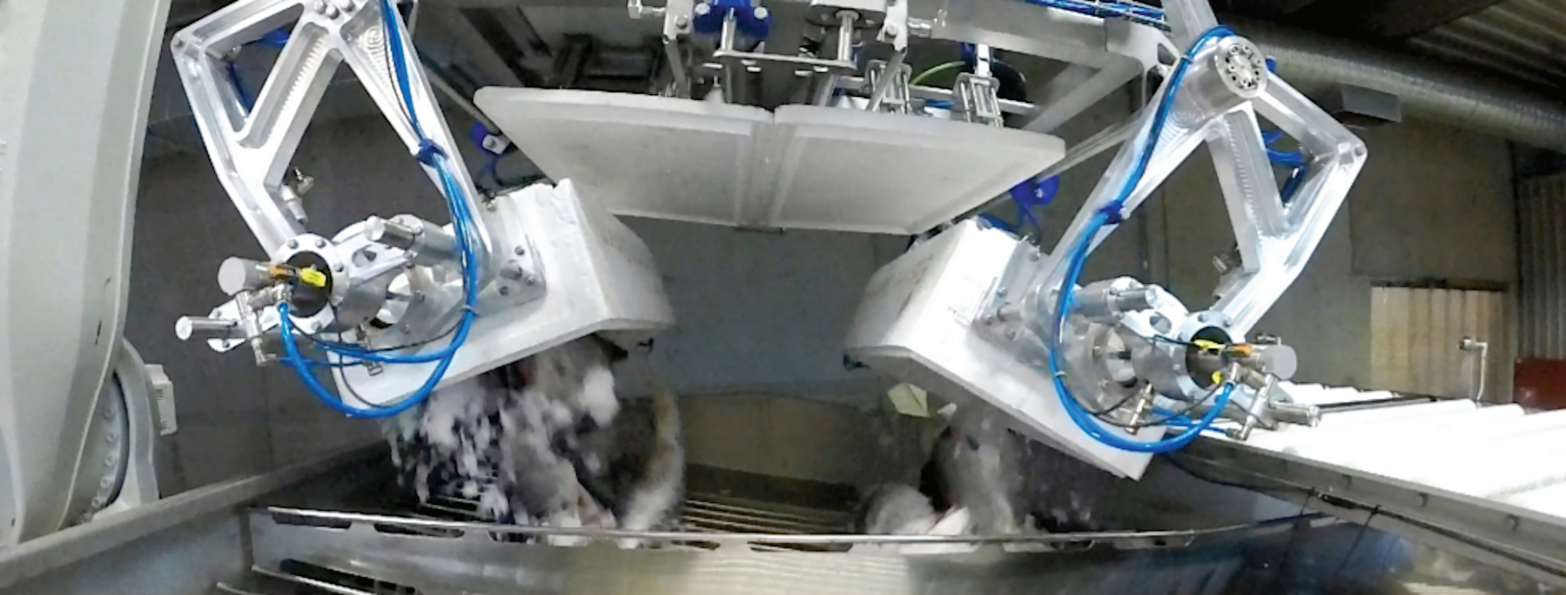
An automated solution for unloading whole salmon from boxes. Courtesy of Marel.

As in many other production industries, automation in fish processing has focused on eliminating dangerous, difficult, and repetitive tasks. Some tasks are ergonomically challenging, such as unloading fish from stacks of large bins into the processing line, also known as de-palletizing. The task of de-palletizing is a relatively simple task to automate, due to the limited variability between containers and boxes on pallets. As the variability is low, a simple machine vision or sensing system can be used to estimate the placement of boxes in relation to the rest of the processing line. Coupling this with a robotic arm, a de-palletizing solution can be achieved (see Figure 3).

**Figure 4.**
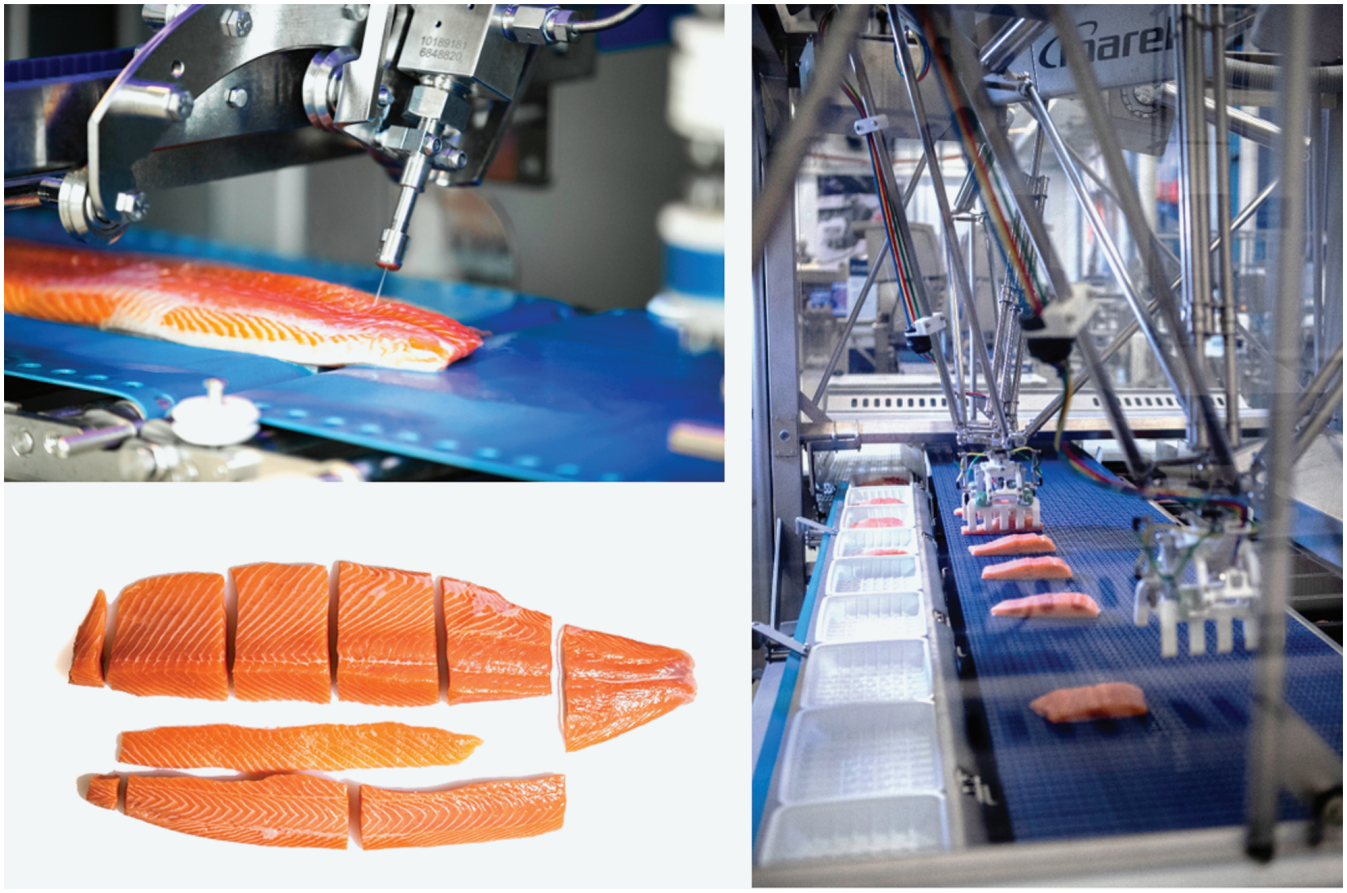
Salmon fillet processing. The pin bones of the fillet are removed with a guided robotic water jet cutter and portioned (top- and lower left pictures) and then picked by an automated gripper off the conveyor and placed into boxes. Courtesy of Marel.

If we consider the other end of the spectrum, automating the trimming of defects in fish fillets requires technology that is more advanced. Defects in fish fillets either can be of natural causes, such as parasites, melanin spots, and disease, or it can be caused by the fishing method and handling of the fish on board the vessel or in the processing hall. This is in addition to defects that can happen when using automatic processing, such as miscuts in filleting machines or inadequate peeling of shrimp. In order to automate the trimming process, a machine would need to mimic a person’s ability to identify defects, determine their severity, and determine a trimming action based on the defect in question. A fish fillet is also a non-rigid structure, so manipulating it requires delicate handling. Another task in the trimming process is the removal of pin bones. Pin bone attachment in salmonoids tends to weaken during post-rigor, making it easier to remove the pin bones manually. Operators then must rely on their sight and hands to locate the bones and remove each one from the fillet, which is a tedious process. This process can be automated using mechanical methods, but doing a proper quality inspection to make sure all bones have been removed is challenging because salmon bones are thin. Over the last decade, X-ray technology has seen a substantial decrease in cost and has been adapted for use in meat plants as a tool for bone detection in the processing line. In the case of many species of fish, for example, cod, the pin bones cannot easily be picked out and are removed by cutting out the area where the pin bones are located. This is also the case with salmon in the pre-rigor mortis stage. Figure 4 shows an example of a machine that locates bones by means of X-ray technology and then automatically cuts the region where the bones are located away with a water jet guided by a motion or robotic system. Such a process has made removing salmon pin bones in a pre-rigor state achievable, which in turn also extends the shelf life of the boneless fillet since the product can go to market 2 to 3 days earlier. The X-ray image data can also be used to estimate density and thereby the thickness throughout the fillet. Based on the volume estimate, portions can be calculated and automatically cut. To optimize the packing of the individual portions, a pick-and-place robot arm can be used to pick pieces off the conveyor and place them into boxes or trays. By automating this step, the piece combinations can match the orders the food producers need to fulfill, creating value by minimizing labor use and reducing give away of the sold packages at the same time.

## Machine Vision

**Figure 5.**
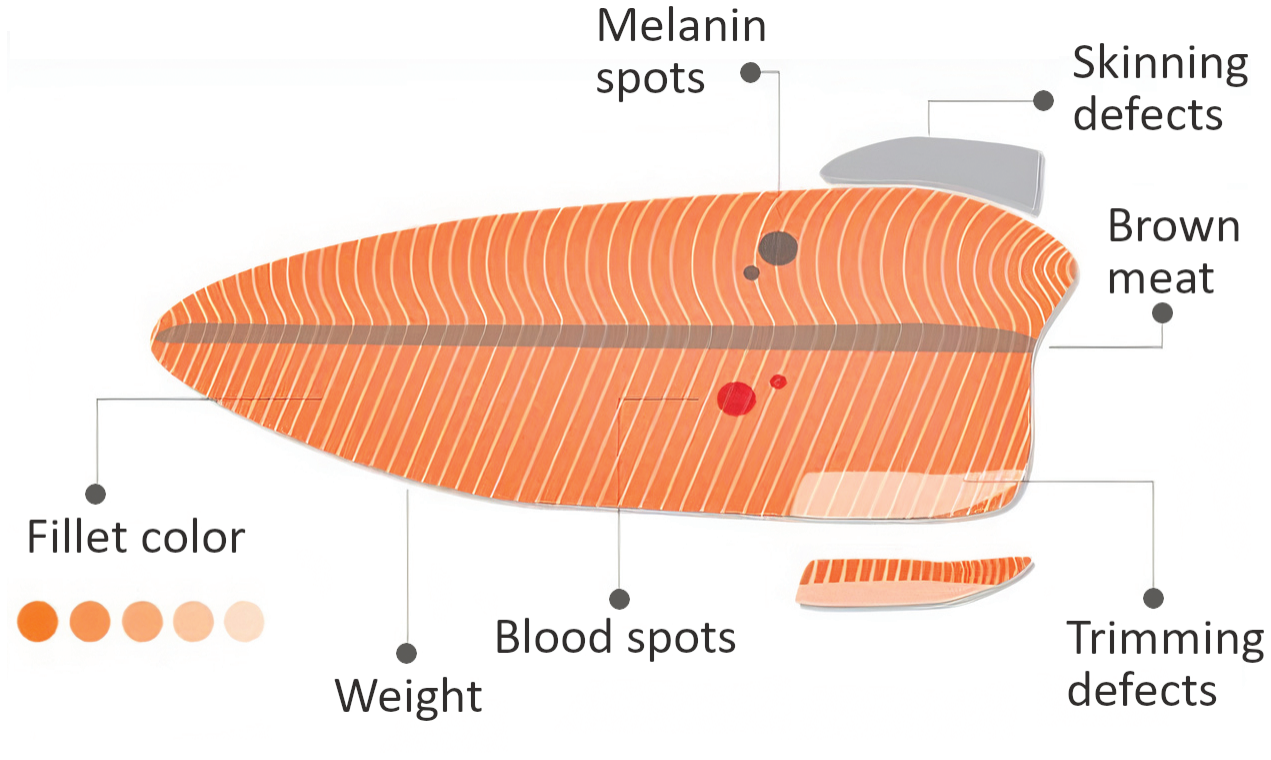
Typical quality defects in salmon fillets. Courtesy of Marel.

In a fish processing line, operators need to be able to identify dark pigment deposits from blood spots and melanin spots, remove brown meat from the fillet center, grade according to a color scale, and remove other skinning defects such as leftover fins and membranes. Operators undergo training to understand quality defects such as those shown in Figure 5. The perceived severity of a defect can vary highly between operators on the trimming line. This variation in perception can cause an unwanted drop in yield, as operators may tend to over-trim fillets to minimize the risk of complaints.

As straightforward as it may seem to automate, for example, bone detection in an X-ray image, a significant effort is placed on algorithm development to automatically detect them (Mery et al., 2011). An algorithm is a series of mathematical equations and sets of rules to determine different aspects of the images such as the presence of bones. 

**Figure 6.**
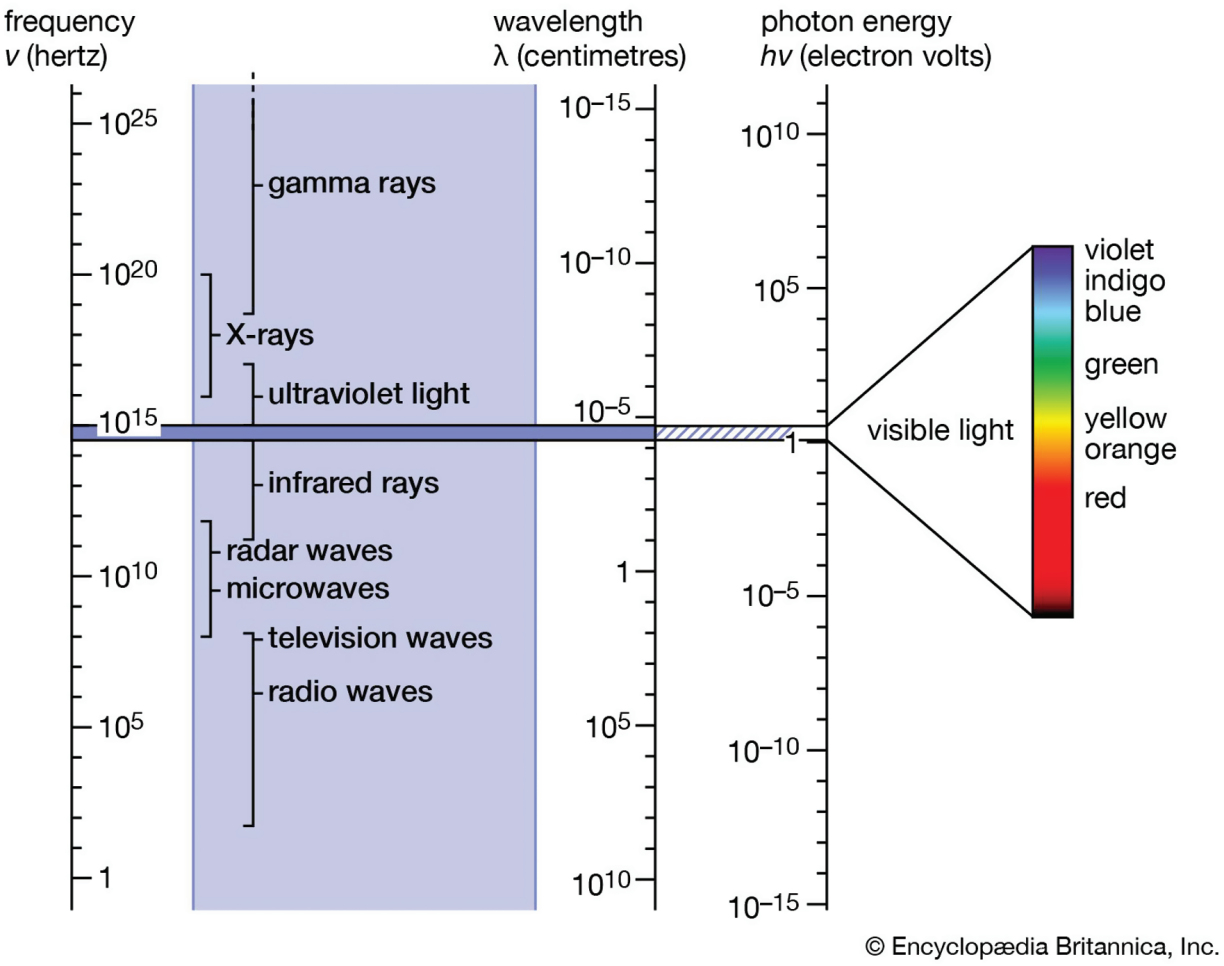
The electromagnetic spectrum. The narrow range of visible light is shown enlarged at the right (Encyclopaedia Britannica, 2022).

For a machine to automatically determine quality defects and remove them, it needs to be able to objectively evaluate the fillet quality. Computer vision has taken leaps in recent years where cameras, sensors, and lighting technologies are applied to mimic human sight (Kopparapu, 2006). Some inspection tasks can be solved by conventional imaging such as color grading, while others require more advanced machine vision technology (Bhargava and Bansal, 2021). To further elaborate, a digital image can capture the same information from a surrounding as humans perceive it. Camera technology can also extend beyond what a person can see. Human sight can only sense a limited part of what is known as the electromagnetic spectrum, as illustrated in Figure 6. This range is known as the visible range, which covers only a narrow part of the spectrum (400 to 700 nm). Beyond the visible range are ultraviolet, infrared, X-ray, and even radio waves and gamma rays. Camera technology today can sense a large part of the spectrum, although each camera category is limited to sensing a specific part. Hyperspectral cameras are becoming commercially available, which can measure multiple narrow bands of the electromagnetic spectrum, essentially giving insight into the chemical composition of what is imaged (Feng and Sun, 2012). These advances in camera technology open new application potentials. Cheng and Sun (2014) illustrated how hyperspectral imaging can be applied to perform quality analysis and control of fish and other seafoods. Another study showed the application of hyperspectral imaging combining visible and near-infrared imaging to differentiate between fresh and frozen-thawed fish fillets (Zhu et al., 2013). By developing solutions that can sense this wide range, image information can be obtained to discriminate between muscle tissues, fat stripes, membranes, and blood spots (Menesatti et al., 2010; Heia et al., 2012; Cheng and Sun, 2014). With advances in imaging technologies, the potential where machine vision can automate inspection tasks for quality control and food safety is numerous.

## Artificial Intelligence

**Figure 7.**
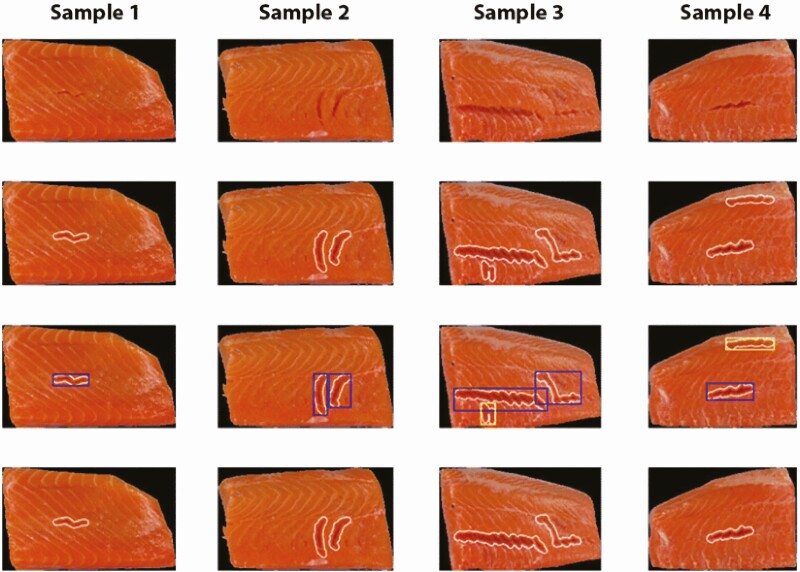
Visualization of results obtained from a deep learning model trained to identify gaping in salmon fillets (Xu and Sun, 2018).

Just as eyesight is not enough for a human to interpret a scene, having a camera system for imaging is not sufficient to solve an automated inspection task. Our adept ability to evaluate our surroundings and make decisions based on the visual input is supported by our brain’s capability to process and decode that input. Similarly, images require computers to further analyze and evaluate different attributes in an image. Conventionally, image analysis has relied heavily on contrasts in colors and brightness in images along with differences in patterns. A classical image analysis approach to determine whether an image contains a face can be done by applying simple pattern analysis to determine the likelihood of the image containing eyes, nose, and mouth. Feature engineering is the field in which a computer vision expert will fine-tune algorithms to be able to determine well-defined features in images. With advances in increased computation power and prices of computers rapidly declining, new approaches to image interpretation have come to light. A disruptive trend in the past decade is known as deep learning (Kakani et al., 2020). Deep learning is a subcategory within machine learning and artificial intelligence (AI) that imitates how humans gain their knowledge. Deep learning can be applied to perform predictive modeling to, for example, determine the presence of breast cancer in mammograms (Yala et al., 2019). Instead of tedious work to hard-code rules and calculations to determine different image properties, deep learning can abstract information from labeled images. The main difference in hardware required for the traditional image analysis approach, and deep learning is the need for a powerful processing unit. As Kakani et al. (2020) highlight, the introduction of graphical processing unit (GPU) has enabled a different approach to image analysis, and rather than relying on detailed feature engineering work, an AI model can be trained to understand image properties based on massive amounts of labeled data. AI models are capable of achieving tasks such as image classification (is there a human in an image), object detection (where are the eyes of the human located precisely in the image), and even segmentation (what regions of the image contain hair) (Guo et al., 2016). Major companies such as Google, Amazon, and Facebook have been basing their success on AI. These companies have created numerous consumer products driven by their AI research (Markoff, 2016; Pan, 2016). Almost any camera app today will contain an image-enhancing feature based on AI, be that an augmenting face filter or scene enhancement. Self-driving cars even rely on AI to determine surroundings such as pedestrians and traffic signs (Daily et al., 2017). Fish processing has been no exception in adapting computer vision and AI methodologies to enable new automation applications. A recent study presented how deep learning can be applied to automatically determine individual fish size from echosounder equipment to reduce the catch of undersized fish in commercial trawling (Garcia et al., 2019). Another example where deep learning has been applied is the detection of salmon muscle gaping as shown in Figure 7, a quality defect affecting the end quality and grade of a salmon fillet (Xu and Sun, 2018). Solving the tasks of detecting gaping by a classical image analysis approach would require a major feature engineering effort. By applying deep learning, the task is reduced to labeling a set of images manually and training the algorithm to identify gaping regions. Vision technology and AI has also been used in shellfish processing to identify pieces of shell after peeling of shrimps. 

### Advanced and Intelligent Robotics

With increasing capabilities to sense and analyze products moving on a processing line, it becomes possible to start automating complex tasks with mechatronics (the combination of mechanical and electronic components) and robotics. Manufacturing industries have seen significant advances within robotics in recent years, and a prime example is how warehousing has been completely transformed with the introduction of mobile robots. For example, an Amazon warehouse has dozens of self-driving robots navigating around the floor, collecting products from shelves, and delivering them to packaging stations for further transport to consumers (Eppner et al., 2016). Similarly, the healthcare industry is starting to incorporate technologies such as surgical robots to perform high-precision surgeries on patients. When looking at these advances one may ask why robotics has still not completely transformed the food production industry. To give context, the surgical robots are taken as an example. Actual surgeons operate the surgical robots, and the robots are, therefore, merely a tool to the surgeon. They are used to optimize precision and to improve sanitation and hygiene during operation. Surgical robots can, therefore, be considered as a remote extension to the surgeon, and fully automating the operation of surgical robots still requires significant developments (Svoboda, 2019). In addition, a surgery may take several hours to perform successfully on a single patient. The throughput required for food production is at a completely different scale. A poultry plant can process over 2 million birds per week (Alonzo, 2021), putting extreme requirements on processing speed. For a salmon factory, it is not unusual that the process from receiving a fish into the processing line until final packaging takes under 5 min. Still, there remains the demand for accuracy and yield in the food processing industry. Although surgical precision is not required, any yield loss is value lost for food producers and an unnecessary source of food waste in the supply chain. Deploying robotic solutions into food production requires solutions capable of achieving the same accuracy as human operators, at speeds surpassing human capability. Several technology trends are creating stepping-stones toward a fully automated future, such as reinforcement learning (Kalashnikov et al., 2018, preprint) and advanced gripper technology (Low et al., 2021). Reinforcement learning is a methodology used to train machine learning models to optimize (e.g., the machine’s performance). Gripper technology is the design of tooling that can manipulate food products, such as picking products from a conveyor and placing in a tray. Gripping technology today often tries to resemble elements from nature, such as suction cups on octopuses’ arms or grasping capabilities of human hands. Other advances that are on the horizon are mobile robots (Gao et al., 2018; Rubio et al., 2019) and collaborative robots, capable of performing in the harsh food production environment. To be able to deploy collaborative robots capable of performing alongside human operators will open enormous opportunities for tasks such as loading of raw materials into the production line. Furthermore, mobile robots could potentially play an important part in transforming logistics in food production plants by transporting products between destination points. A recent publication illustrated how collaborative robots could be applied to handle food products that are non-rigid, which is essential to perform operations such as trimming and handling of fish fillets (Long et al., 2021).

### Future Outlook

One might ask what the food production plant of the future will look like? Can a lights-out manufacturing utopia be achieved within the next few years or so? To reach this milestone, there are still quite some challenges ahead, such as further developing intelligent machines and advancing sensing technology capabilities. As prices decrease for advanced technologies, they become more applicable in industrial settings, providing further opportunities to automate production plants. A robotic solution capable of trimming quality defects from fish fillets with minimal yield loss is perhaps not too far on the horizon, and it is merely a question of when rather than if this step in the fish processing will be automated. To achieve this goal, knowledge transfer from academia to industry is essential to incorporate advanced technologies into future food production solutions.

## About the Authors

**Bergur Gudmundsson** has BSc honors in fisheries science. His specialty is optimization of the seafood value chain with emphasis on productivity and business results. He is currently an Industry Sales Manager for new business areas at Marel Fish.

**Hildur Einarsdottir** holds a PhD in Applied Mathematics and Computer Science and an MSc in Electrical Engineering. She specializes in computer vision, image and data analysis, and project management. Currently, she is a program manager at Marel focusing on Automation and introducing new technologies into the product portfolio of the company.

**Validmar Ómarsson** holds an MSc in Mechatronics, Robotics, and Automation Engineering. His specialties lie within combining technical expertise with business understanding to bring new automation solutions to the food production industry. His current role is Director of Innovation at Marel Fish.
